# Comprehensive analysis of cystatin family genes suggests their putative functions in sexual reproduction, embryogenesis, and seed formation

**DOI:** 10.1093/jxb/eru274

**Published:** 2014-07-04

**Authors:** Peng Zhao, Xue-mei Zhou, Jie Zou, Wei Wang, Lu Wang, Xiong-bo Peng, Meng-xiang Sun

**Affiliations:** ^1^Department of Cell and Developmental Biology, College of Life Sciences, State Key Laboratory of Plant Hybrid rice, Wuhan University, Wuhan 430072, China; ^2^Molecular Genetics Key Laboratory of China Tobacco, Guizhou Academy of Tobacco Science, Guiyang 550081, China; ^3^Key Laboratory of Plant Germplasm Enhancement and Specialty Agriculture, Wuhan Botanical Garden of the Chinese Academy of Sciences, Wuhan 430074, China

**Keywords:** Cathepsin L-like proteases, cystatin, embryogenesis, seed development, sexual reproduction, tobacco.

## Abstract

Cystatins are tightly bound and reversible inhibitors of cysteine proteases in C1A and C13 peptidase families, which have been identified in several species and shown to function in vegetative development and response to biotic/abiotic stresses in plants. Recent work revealed their critical role in regulating programmed cell death during embryogenesis in tobacco and suggested their more comprehensive roles in the process of sexual plant reproduction, although little is known about cystatin family genes in the processes. Here, 10 cystatin family genes in *Nicotiana tabacum* were identified using an expressed sequence tag (EST)-based gene clone strategy. Analysis of their biochemical properties showed that nine of them have the potency to inhibit the activities of both commercial cathepsin L-like proteases and extracted cysteine proteases from seeds, but with different *K*_i_ values depending on the types of proteases and the developmental stages of the seed tested. This suggests that cystatin-dependent cathepsin L-like proteolytic pathways are probably important for early seed development. Comprehensive expression profile analysis revealed that cystatin family genes showed manifold variations in their transcription levels in different plant cell types, including the sperm, egg, and zygote, especially in the embryo and seed at different developmental stages. More interestingly, intracellular localization analysis of each cystatin revealed that most members of cystatin families are recognized as secretory proteins with signal peptides that direct them to the endoplasmic reticulum. These results suggest their widespread roles in cell fate determination and cell–cell communication in the process of sexual reproduction, especially in gamete and embryo development, as well as in seed formation.

## Summary

The survey of expression patterns, biochemical characters, and intracellular localizations of cystatins in tobacco reveals their widespread roles in gamete development, embryogenesis, and seed formation.

## Introduction

Cystatins are tightly bound and reversible inhibitors of papain-like and legumain-like proteases, which have been identified in vertebrates, invertebrates, plants, and other organisms. Notably, cystatins in plants form an independent subfamily clustering in a branch distinct from other cystatin families on the phylogenetic tree (Margis *et al.*, 1998). Most cystatins in plant have a molecular mass in the 12–16kDa range (Misaka *et al.*, 1996; Martinez *et al.*, 2005), and a few of them have a molecular mass of ~23kDa due to a C-terminal extension, which contributes to the inhibition of legumain-like protease activities (Martinez *et al.*, 2007). Cystatin family genes have been predicted in several model plants with known completed genome sequences, such as *Oryza sativa*, *Arabidopsis thaliana*, and *Populus trichocarpa* (Martinez *et al.*, 2005; Martinez and Diaz, 2008), or without genome information, such as *Hordeum vulgare* (Martinez and Diaz, 2008). However, only a few of them have been well characterized and shown to function in several physiological processes in plants, including programmed cell death (PCD) (Solomon *et al.*, 1999; Zhao *et al.*, 2013), seed germination (Hwang *et al.*, 2009), and defence mechanisms against pathogens (Gutierrez-Campos *et al.*, 1999; Belenghi *et al.*, 2003), insect attack (Goulet *et al.*, 2008; Konrad *et al.*, 2008), and abiotic environmental stresses (Hwang *et al.*, 2010), but their relationship to the processes of sexual reproduction, embryogenesis, and seed formation is largely unknown.

The main target of cystatin is the cysteine proteases in the peptidase C1A family, which are usually synthesized as inactive precursors comprised of an N-terminal signal peptide and the mature protein. Cysteine proteases in subfamily C1A in plants are divided into four groups, cathepsin B-, H-, F-, and L-like, according to their closest animal counterparts (Martinez and Diaz, 2008). The activities of the cysteine proteases *in vivo* may be controlled by several mechanisms, including local zymogene concentration and the presence of a specific repertoire of cystatin inhibitors (Cambra *et al.*, 2012), which have also been reported to function in various physiological processes, such as pollen development (Lee *et al.*, 2004; Zhang *et al.*, 2009), senescence (Eason *et al.*, 2005), tracheary element microautolysis (Avci *et al.*, 2008), and defence against pathogens (Kruger *et al.*, 2002; Gilroy *et al.*, 2007), indicating that the proteolytic pathway of cystatin-dependent cysteine proteases is crucial to many physiological processes in plant development.

Although peptidase–inhibitor interactions are crucial to several important processes in plant development as described above, the roles of cystatin and their targets in some significant processes of sexual plant reproduction including gametogenesis, embryo development, and seed formation are largely unknown due to the technical limitation of collecting gametes and early embryos. *Nicotiana tabacum*, as a very important commercial crop worldwide, has been considered to be an ideal model plant for the study of tissue culture (Murashige and Skoog, 1962), genetic engineering (Horsch *et al.*, 1985), embryogenesis (He *et al.*, 2007), and host–pathogen interactions (Thara *et al.*, 2004). Ten years ago, it was found to be possible to isolate and collect living sperm, egg cells, zygotes, and embryos of tobacco successfully, and cell type-speciﬁc cDNA libraries have been constructed in recent years (Ning *et al.*, 2006; Ma *et al.*, 2011; Xin *et al.*, 2011; Zhao *et al.*, 2011). Therefore, it is now possible to conduct a comparative bioinformatics, biochemical, and expression profile analyses of cystatin family genes in those significant processes of sexual plant reproduction to provide valuable insights into the roles of the cystatin-dependent proteolytic pathway in these processes. Thus, an expressed sequence tag (EST)-based method was used to identify novel cystatins in tobacco, and a comprehensive analyses was also carried out to gain insight into their putative roles in the sexual reproductive process, especially in the process of gamete development, embryogenesis, and seed formation.

## Materials and methods

### Plant materials

*Nicotiana tabacum* L. cv. Petite Havana SR1 plants were grown under 16h/8h light/dark cycles, at 25 °C in the greenhouse.

### Identification of cystatins in tobacco

A total of 3.5×10^5^ EST sequences in tobacco were collected from GeneBank and constructed into a local BLAST database. The tBlastn program using conserved protein sequences of cystatin in *Arabidopsis thaliana* was run, and EST sequences related to cystatin genes were collected. EST assembly was executed using the ContigExpress program, with a minimum of 80% identity in the overlap region and a minimum overlap of 50 bases. After assembly, redundant sequences were removed manually, and groups that contained only one EST sequence were classiﬁed as singletons. Open reading frame (ORF) analysis of each contig was performed using OMEGA, and the BLASTP program of the National Center for Biotechnology Information (NCBI) with intact or partial deduced protein sequences of each contig. The contigs with a partial or intact cystatin domain based on information obtained were selected as candidates for further study.

### Isolation of full-length cDNA of each cystatin in tobacco

After ORF analysis, full-length cDNA of each contig was obtained through the rapid amplification of cDNA ends (RACE) approach. Full-length sequences were confirmed by reverse transcription–PCR (RT–PCR) with specific primers at the 5’ and 3’ end, respectively (Supplementary Table S1 available at *JXB* online). RT–PCR was carried out in a 50 μl PCR mixture containing 5 μl of 10× Ex *Taq* buffer, 2.5mM MgCl_2_, 200 μM dNTPs, 0.2 μM of primers, 1.2U of Ex *Taq* DNA polymerase (Takara), and cDNA prepared from different tissues. Conditions for PCR on the T100™ Thermal Cycle PCR system (Bio-Rad) are as follows: initial denaturation at 94 °C for 2min; 35 amplification cycles with denaturation at 94 °C for 30 s, annealing at *T*_m_ –5 °C for 30 s; extension at 72 °C for 1min; and a final incubation at 72 °C for 5min.

### Protein sequence and phylogenetic analysis

In order to analyse the relationships of cystatin family genes identified in tobacco to other cystatin family genes in other plant species, a multiple sequence alignment of the known cystatin family genes in some model species was conducted with Clustal X ver. 1.81 using the default multiple alignment parameters. The tree was constructed with Phylip Ver. 3.68 using the Protpars method.

Prediction of the signal peptide of each cystatin was performed on the SignalP server (Petersen *et al.*, 2011). The secondary and three-dimensional structures of each cystatin were predicated on the PSIPRED v3.3 server (Buchan *et al.*, 2010) and SWISS-MODEL workspace (Arnold *et al.*, 2006; Kiefer *et al.*, 2009), respectively. Conserved motifs among tobacco cystatins were analysed on http://weblogo.berkeley.edu/.

### RNA isolation and RT–qPCR

Total RNAs of leaf, root, stem, anther, pistil, petal, sepal, pollen, and pollen tube were extracted using TRI Reagent Solution (Ambion), and total RNAs of seeds at different stages were extracted with RNAqueous™ (Ambion). All total RNAs were treated with RNase-free DNase I (Promega) and cDNAs were synthesized using ReverTra Ace (Toyobo) under the conditions recommended by the manufacturer. mRNA isolation from sperm cells, egg cells, zygotes, apical cells, basal cells, and embryos at different stages and cDNA synthesis were performed according to a previous procedure (Ma *et al.*, 2011; Xin *et al.*, 2011; Zhao *et al.*, 2011). Quantitative real-time reverse transcription–PCR (RT–qPCR) was conducted for cystatin gene expression pattern analysis. RT–qPCR was performed in a 20 μl reaction mixture containing 10 μl of 2×FastStart Universal SYBR Green Master (Roche), 250nM of each primer (Supplementary Table S1 at *JXB* online), and cDNA prepared from different tissues. Conditions for RT–qPCR were as follows: activation of FastStart *Taq* DNA polymerase at 95 °C for 10min, and >40 cycles (95 °C for 15 s and 60 °C for 1min) with a Rotor-Gene 6000 system (Corbett Research). The data analysis was conducted according to a previous procedure (Ma *et al.*, 2011).

### Heterologous expression and purification

The coding regions of cystatin family genes lacking the stop codon and signal peptide sequences were cloned (Supplementary Table S2 at *JXB* online) and inserted into the pMXB-10 vector (NEB). The resulting plasmids were transformed into *Escherichia coli* BL21 (DE3) (Novagen). The recombinant cystatins were expressed and purified according to the manufacturer’s instructions. The purified cystatins were re-purified by ion exchange chromatography with a Bio-Scale™ Mini UNOsphere™ Cartridge Q/S or a Bio-Scale™ Mini CHT Type I Cartridge (Bio-Rad) on BioLogic DuoFlow™ system (Bio-Rad). The final protein concentrations were quantified using a Coomassie Plus kit (Thermo) with bovine serum albumin as the standard.

### Inhibitory activities of cystatin against model cysteine proteases and total protein extracts from tobacco seeds

For determination of the *K*_i_ values of the interaction of each cystatin with the model cysteine proteases papain (Sigma-Aldrich), human liver cathepsin L (Sigma-Aldrich), cathepsin B (Sigma-Aldrich), and cathepsin H (Merck), substrate hydrolysis progress curves of each cysteine protease were monitored according to a previous method (Zhao *et al.*, 2013) with or without the addition of recombinant cystatin under reducing conditions.

For the determination of the inhibitory potency of each cystatin against total extracts from seeds at different stages, all samples were frozen in liquid nitrogen and ground in a mortar using a pestle. After grinding, the samples of seeds were suspended in 50mM MES (pH 6.0), 2mM EDTA, 10% glycerol, 0.1% CHAPS, 0.01% Brij-35, 2% polyvinylpolypyrrolidone (PVPP), 10mM l-cysteine, and 10mM sodium metabisulphite. All samples were then incubated on ice for 1h, centrifuged (14 000 *g*, 30min, 4 °C), and the supernatants were collected for further analysis. The final concentrations of total protein in the supernatants were quantified using a Coomassie Plus Kit (Thermo) with bovine serum albumin as the standard.

The inhibitory potency of each recombinant cystatin against total protein from tobacco seeds was tested by monitoring hydrolysis of the substrates Z-FR-AMC (*N*-carbobenzoxyloxy-Phe-Arg-7-amido-4-methylcoumarin), Z-RR-AMC (*N*-carbobenzoxyloxy-Arg-Arg-7-amido-4-methylcoumarin), and Bz-FVR-AMC (benzoyl-Phe-Val-Arg-7-amido-4-methylcoumarin) susceptible to degradation by cathepsin L-, B-, and H-like proteases, respectively. Hydrolysis was allowed to proceed at 30 °C in a 100 μl assay mixture containing 50mM sodium phosphate (pH 6.0), 5 μg of total protein, 25 μΜ substrate, 10mM l-cysteine, 1mM EDTA, and 0.01% Brij-35 with or without the addition of 1 μΜ recombinant cystatins (NtCYS2 1.31 μg, NtCYS3 2.81 μg, NtCYS4 1.51 μg, NtCYS5 1.33 μg, NtCYS6 1.08 μg, NtCYS7 1.08 μg, NtCYS8 1.47 μg, NtCYS9 1.51 μg, and NtCYS10 1.61 μg). The activity levels were monitored using a Spectra Max M2 (Molecular Device Co.) with excitation and emission filters of 360nm and 455nm, respectively.

### Intracellular localization of cystatins in tobacco

For intracellular localization analysis of each cystatin identified in tobacco, *35S::eGFP-NOS* was firstly constructed in pRS300 to generate the pRS300-35S-eGFP-NOS vector. The full-length cystatin coding sequences (without stop codons) were amplified and inserted in-frame with enhanced green fluorescent protein (eGFP) into the vector pRS300-35S-eGFP-NOS to generate *35S::NtCYS-eGFP-NOS* expression vectors (Supplementary Table S2 at *JXB* online). *35S::NtCYS-eGFP-NOS* expression vectors were co-expressed with an endoplasmic reticulum (ER) marker containing an N-terminal signal peptide derived from a vacuolar basic chitinase of *A. thaliana* and the C-terminal amino acid sequence HDEL (RFP-ER) (Haseloff *et al.*, 1997), and the Golgi marker ST-RFP (a fragment of a rat α-2,6-sialyltransferase fused to red fluorescent protein) (Saint-Jore *et al.*, 2002) in *Allium cepa* epidermal cells through particle-mediated transient transformation using a PDS-1000/He instrument (Bio-Rad, USA). Coating by gold particles and bombardment were performed according to the manufacturer’s instructions (Bio-Rad Laboratories). Transformed *A. cepa* epidermis was observed under a confocal microscope (Olympus FluoView FV1000). Images were processed with Adobe Photoshop.

## Results

### Collection and identification of cystatin family genes in tobacco

To identify cystatin family genes in tobacco, 3.5×10^5^ EST sequences from tobacco were downloaded from the database at the NCBI, and constructed into a local BLAST database. An tBLASTn search was carried out using conserved protein sequences of the cystatin family, and an expectation value <1.0×10^–5^ was considered to indicate true cystatin family genes. A total of 119 EST sequences related to cystatin family genes were obtained and assembled into 15 contigs, and redundant sequences were omitted manually. The full length of these sequences was obtained through the RACE technique, and detailed information on each gene is given in Table 1. ORF analysis indicated that each gene contains a complete ORF of 294–753 nucleotides. BLASTP searches with the deduced protein sequences of the predicted cystatin genes returned several matches with proteins containing cystatin domains, indicating that these predicted genes are new members of the cystatin family in *N. tabacum*, including a known cystatin *NtCYS1*. Thus, the others genes were designated in numerical sequence.

**Table 1.** Detailed information on cystatin family genes in tobacco

Cystatin	ORF (bp)	Predicated protein information						
		No. of amino acids	Signal peptide	Signal peptide length (aa)	Mol. wt (kDa)	pI	α-Helix	β-Strand
NtCYS1	420	140	+	27	15.3	6.71	1	5
NtCYS2	360	120	+	28	13.1	9.56	1	5
NtCYS3	753	251	+	25	28.1	5.69	2	12
NtCYS4	408	136	+	21	15.1	9.77	1	4
NtCYS5	366	122	+	28	13.3	10.01	1	5
NtCYS6	294	98	–	–	10. 8	5.83	1	5
NtCYS7	294	98	–	–	10.8	5.43	1	5
NtCYS8	393	131	+	27	14.7	7.77	1	5
NtCYS9	402	134	+	24	15.1	8.53	1	5
NtCYS10	426	142	+	29	16.1	9.10	1	4

**Fig. 1.**
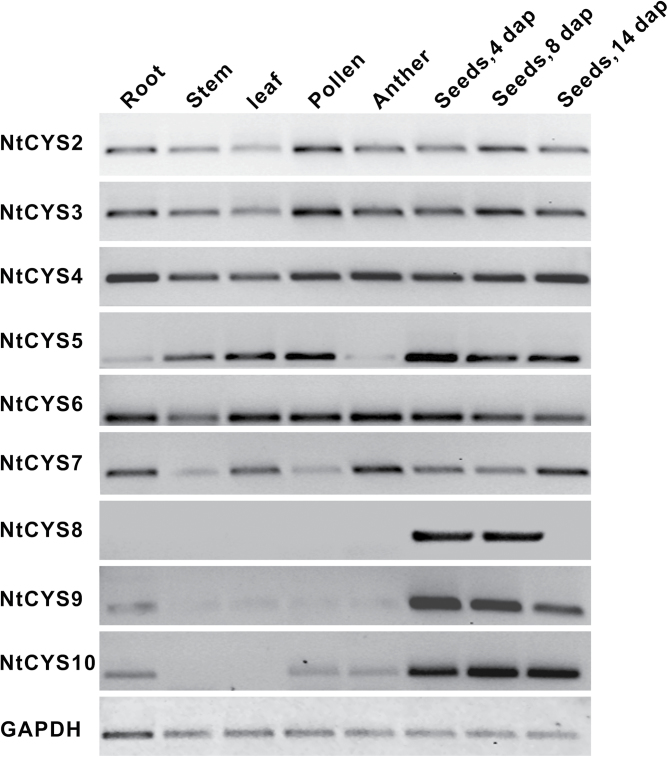
RT–PCR examination of the transcripts of novel cystatin family genes in tobacco. cDNA prepared from root, stem, leaf, pollen, anther, and seeds at stage 1, 5, and 9 were selected as templates for PCR. Glyceraldehyde-3-phosphate dehydrogenase (GAPDH) was used as the control. The nine stages of seed development were divided according to the corresponding stages of embryogenesis in tobacco (Zhao *et al.*, 2013).

To confirm further the existence of the predicted cystatin genes in tobacco, cDNA prepared from different tissues including leaf, stem, root, pollen, anther, and seeds at stages 1, 5, and 9 were selected as templates for RT–PCR. PCR parameters were optimized to identify cystatin genes in these tissues: 28 cycles for the housekeeping gene *GAPDH* (glyceraldehyde-3-phosphate dehydrogenase) and 35 cycles for cystatin genes. The transcripts of nine novel predicted cystatin genes can be detected in different tissues of tobacco as shown in Fig. 1. Interestingly, all of them can be detected in seeds at early stages (stage 1 and stage 5). The transcript of *NtCYS8* is only detected in early seeds, but not in the other tissues tested, indicating its specific roles in early seed development. These data suggest that all predicted novel cystatin genes exist in tobacco, and display different expression patterns, implying their specific roles in different stages of tobacco development.

### Protein sequence and phylogenetic analysis

**Fig. 2.**
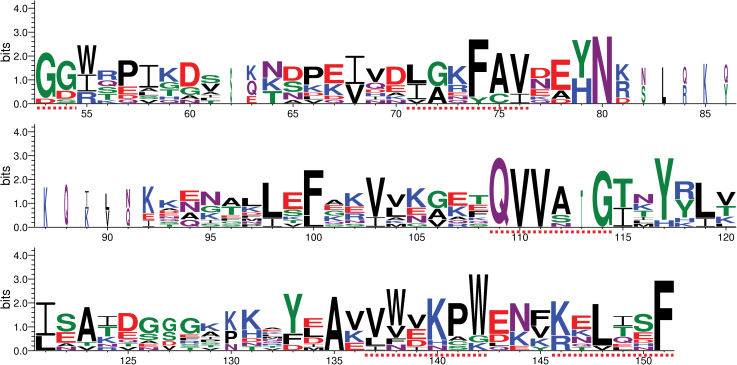
Conserved motifs in tobacco cystatins. Conserved motifs in cystatins are labelled with dotted lines, and the number under the character of amino acids indicates the position of amino acids in the protein sequences corresponding to that in Supplementary Fig. S1 at *JXB* online. (This figure is available in colour at *JXB* online.)

As described above, 10 cystatin family genes including nine novel genes have been identified in *N. tabacum*. The sequence features and functional motifs of each of the cystatins were then investigated. Among them, nine cystatins have a molecular mass in the range of 10.8–16.1kDa, and only NtCYS3 has a molecular mass of ~28.1kDa with a C-terminal extension. Prediction of the signal peptide using SignalP 4.0 (Petersen *et al.*, 2011) shows that eight of these proteins contain a predicted signal peptide, with the two exceptions, NtCYS6 and NtCYS7 (Table 1), indicating that the majority of cystatins in tobacco could enter the endomembrane system and then be secreted into their target compartments. The entire amino acid sequences of the 10 tobacco cystatins were aligned and compared. Some conserved motifs have been identified (Fig. 2; Supplementary Fig. S1 at *JXB* online): (i) one or two glycines at the N-terminus are conserved (except in NtCYS8); (ii) a ‘LARFAV’ motif is present with related substitutions in all proteins (except in NtCYS10); (iii) the active site ‘QxVxG’ is essentially conserved, although an additional amino acid was found in NtCYS8 (‘QVVATG’); (iv) a tryptophan is conserved in the C-terminus of most cystatins, with the exception of NtCYS1 and NtCYS8; (v) two novel motifs, ‘VWxKPW’ and ‘KxLxxF’, were found in the C-terminus of all cystatins with related substitutions; and (vi) a C-terminal extension with ‘SNSL’ was detected in NtCYS3, which is a putative site for the inhibition of the activities of legumain-like proteases.

To compare the similarities and differences of the structures of these cystatin proteins in tobacco, the secondary structures of cystatins were predicated using PSIPRED v3.3 (Buchan *et al.*, 2010) and three-dimensional structures were predicated on SWISS-MODEL workspace using an automated model (Arnold *et al.*, 2006; Kiefer *et al.*, 2009). Most cystatins, with the exceptions of NtCYS3, NtCYS4, and NtCYS10, show similar secondary structures (with one α-helix and five β-strands) and their three-dimensional structures are similar to that observed in rice OC-I cystatin (Table 1; Supplementary Fig. S2 at *JXB* online). The ‘QxVxG’ reactive site is located in the loop between the second and third β-strand of most cystatins, with the exceptions of NtCYS3, NtCYS4, and NtCYS10. The three-dimensional structure of the extended C-terminus of NtCYS3 is different from that of other typical cystatins, with two α-helices and 12 β-strands, which suggests that the changes in three-dimensional structure may contribute to their specific biochemical properties.

**Fig. 3.**
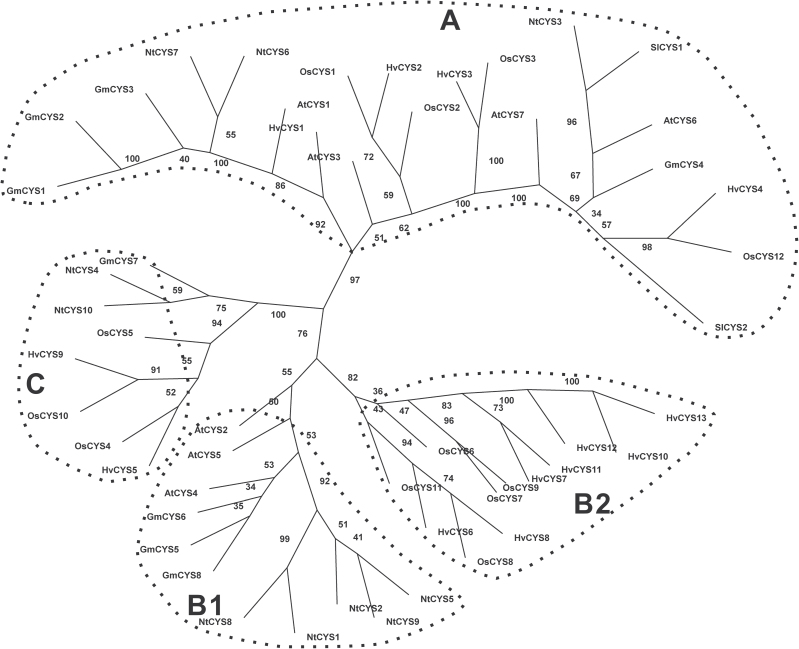
Phylogenetic relationships of the cystatins from *Nicotians tabacum*, *Arabidopsis thaliana*, *Glycine max*, *Oryza sativa*, *Hordeum vulgare*, and *Solanum lycopersicum*. The tree was calculated with Phylip Ver. 3.68 software using the Protpars method. The numbers at the nodes indicate the bootstrap values. Accession numbers of the protein sequences used in this analysis were as follows: *A. thaliana* AtCYS1 (AT5G12140), AtCYS2 (AT2G31980), AtCYS3 (AT3G12490), AtCYS4 (AT4G16500), AtCYS5 (AT5G47550), AtCYS6 (At3g12490), AtCYS7 (At5g05110); *G. max* GmCYS1 (ACU14306), GmCYS2 (CAI84599), GmCYS3 (CAI84598), GmCYS4 (BAA19610), GmCYS5 (ACU19522), GmCYS6 (ACU14962), GmCYS7 (CAI84604), GmCYS8 (CAI84601); *O. sativa* OsCYS1 (Os01g58890), OsCYS2 (Os05g41460),OsCYS3 (Os05g33880), OsCYS4 (Os01g68660), OsCYS5 (Os01g68670), OsCYS6 (Os03g11180), OsCYS7 (Os03g11170), OsCYS8 (Os03g31510), OsCYS9 (Os03g11160), OsCYS10 (Os04g28250), OsCYS11 (Os09g08100), OsCYS12 (Os01g16430); *H. vulgare* HvCYS1 (Y12068), HvCYS2 (AJ748337), HvCYS3 (AJ748338), HvCYS4 (AJ748344), HvCYS5 (AJ748340), HvCYS6 (AJ748341), HvCYS7 (AJ748345), HvCYS8 (AJ748343), HvCYS9 (AJ748339), HvCYS10 (AJ748342), HvCYS11 (AJ748346), HvCYS12 (AJ748347), HvCYS13 (AJ748348); and *S. lycopersicum* SlCYS1 (AAF23126), SlCYS2 (ABG23376).

In order to evaluate the evolutionary relationship among the cystatin proteins, a multiple sequence alignment of the known cystatin family genes from some model plants was conducted using Clustal X ver. 1.81. The phylogenetic tree was constructed with Phylip Ver. 3.68 using the Protpars method. These proteins were clustered into three major groups (A, B, and C) (Fig. 3). Group B is the largest group among them, and comprises two subgroups, B1 and B2. Cystatins from dicotyledons were grouped into subgroup B1, whereas subgroup B2 is comprised of cystatins from monocotyledons. The majority of cystatins in tobacco (NtCYS1, NtCYS2, NtCYS5, NtCYS8, and NtCYS9) fall into group B1. Three tobacco cystatins (NtCYS3, NtCYS6, and NtCYS7) were grouped into group A, and only two cystatins, named NtCYS4 and NtCYS10, were in group C.

### Inhibitory potency of recombinant cystatins against model cysteine proteases

**Fig. 4.**
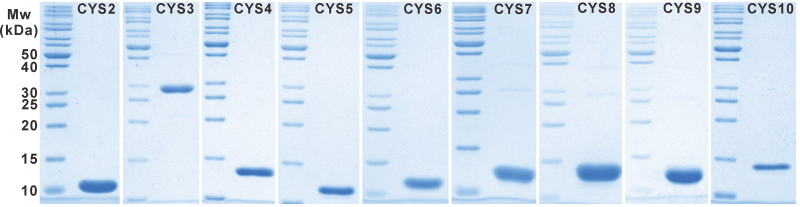
Purification of tobacco cystatins. Coomassie blue-stained SDS–polyacrylamide gel showing purified cystatins.

Most cystatins can inhibit the activities of cysteine proteases in the peptidase C1A family (Arai *et al.*, 2002), and only a few of them can also inhibit the activities of cysteine proteases in the peptidase C13 family (Martinez *et al.*, 2007). In order to investigate the potential inhibitory properties of cystatins in tobacco, recombinant cystatins were successfully expressed in *E. coli* using the IMPACT™ expression system (NEB) and purified in a soluble form. Recombinant proteins of the expected size, free of protein contaminants, were obtained through affinity purification and ion exchange chromatography (Fig. 4). Four model cysteine proteases, namely papain (papaya latex), human liver cathepsin L, cathepsin B, and cathepsin H were chosen for the inhibition assays of recombinant cystatin proteins *in vitro*. As expected, most recombinant cystatins have the potency to inhibit the activities of cysteine proteases, except NtCYS8 (Table 2). Thus, it was futher confirmed that the predicted novel cystatin genes are indeed new members of the cystatin family in tobacco. However, they show different *K*_i_ values for the targeted cysteine proteases depending on the types of proteases tested (Table 2). Generally, all of them primary inhibit cathepsin L-like protease (estimated *K*_i_ values of 10^–10^–10^–12^ M for cathepsin L and 10^–9^–10^–12^ M for papain) followed by cathepsin H (estimated *K*_i_ value of 10^–7^–10^–11^ M). NtCYS4 is the strongest inhibitor for cathepsin L with *K*_i_ values of 3.4×10^–12^ M. NtCYS5 is the strongest inhibitor for papain and cathepsin H, with *K*_i_ values of 9.3×10^–12^ M and 5.1×10^–11^ M, respectively, whereas NtCYS10 is the strongest inhibitor for cathepsin B with *K*_i_ values of 6.4×10^–9^ M. NtCYS8 with the non-typical reactive site ‘QVVATG’ is a putative inhibitor with no visible inhibitory potency for all the cysteine proteases tested, suggesting that the typical reactive site ‘QxVxG’ may play important roles in their biochemical function.

**Table 2.** *K*_i_ values of different cystatins against cysteine proteasesEach value is the mean of three independent experiment ±SD. Three different concentrations of each cystatin were applied in each experiment. No inhibitory effect (Ni) was considered for inhibition <10% at 1 μM of each recombinant cystatin.

Cystatin	*K*_i_ (M)			
	Papain	Cathepsin L	Cathepsin B	Cathepsin H
NtCYS2	1.6±0.4×10^–9^	3.2±0.8×10^–11^	2.1±0.4×10^–7^	4.3±0.1×10^–10^
NtCYS3	3.5±1.3×10^–10^	3.6±0.8×10^–11^	Ni	2.5±0.6×10^–7^
NtCYS4	5.4±2.3×10^–11^	3.4±0.9×10^–12^	1.2±0.4×10^–7^	8.9±1.5×10^–7^
NtCYS5	9.3±2.1×10^–12^	4.7±1.0×10^–12^	3.1±0.5×10^–8^	5.1±0.8×10^–11^
NtCYS6	3.2±1.7×10^–10^	3.7±1.0×10^–11^	Ni	6.7±1.2×10^–9^
NtCYS7	6.3±2.8×10^–10^	1.5±0.1×10^–10^	Ni	8.3±0.5×10^–9^
NtCYS8	Ni	Ni	Ni	Ni
NtCYS9	4.0±0.8×10^–10^	4.5±0.5×10^–11^	2.2±0.1×10^–6^	1.7±0.3×10^–10^
NtCYS10	1.6±0.6×10^–10^	7.2±1.1×10^–12^	6.4±1.9×10^–9^	1.3±0.1×10^–7^

### The expression profiles of cystatin family genes in tobacco

**Fig. 5.**
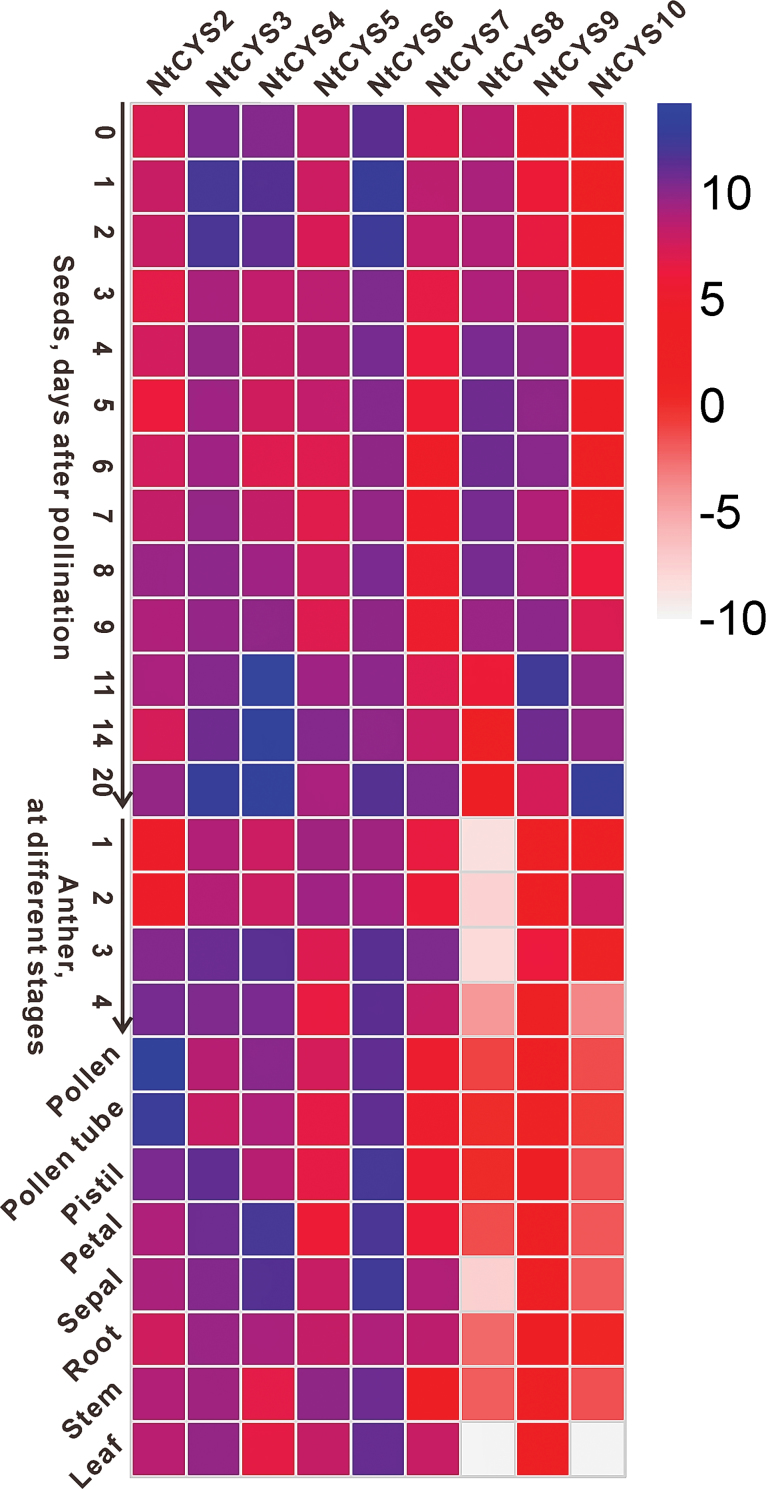
Expression profile of cystatin family genes in tobacco, which was constructed based on the relative expression level of each cystatin gene in different tissues. The expression level was normalized to the average expression level of GAPDH (AJ133422), polyubiquitin (GQ281244), and elongation factor 1α (AF120093). A blue box indicates a higher expression level of the cystatin family genes, whereas a white box indicates a lower expression level of the cystatin family genes. Anthers at stages 1–4 correspond to anthers containing microspore mother cells, tetrads, single-nucleated pollen, and bi-nucleated pollen, respectively. The scale bar represents the fold change (log2 value).

To build the expression profile of cystatin family genes in tobacco, RT–qPCR experiments were carried out based on the cDNA prepared from different cells or tissues such as leaf, stem, root, petal, sepal, anthers at different developmental stages, pollen, pollen tube, pistil, ovule, and seeds at different developmental stages. Heatmap analysis based on the relative expression level of each cystatin gene was performed, and an overview of the expression profile of cystatin genes is presented in Fig. 5. Most cystatin genes exhibited a rather broad expression proﬁle, with the exception of *NtCYS8*, *NtCYS9*, and *NtCYS10*, which were expressed at a relatively low level or were undetectable in most of the vegetative tissues tested (Fig. 5). The heatmap analysis results demonstrate that most cystatin genes are active in reproductive cells or organs including pollen, pollen tube, ovule, and seeds at different stages. In particular, some of them are specifically or abundantly expressed in seeds at some early stages, which may be important for controlling stage-speciﬁc developmental events during seed development.

### The transcription levels of cystatin family genes show manifold variations in sperm cell, egg cell, and zygote

An overview of the expression profiles of cystatin genes in tobacco suggested that cystatin family genes may play important roles in different aspects of sexual reproduction. Fertilization is one of the key processes of sexual reproduction. Early reports showed that the gene expression programmes of the parental gametes play important roles in zygote development (Ning *et al.*, 2006; Autran *et al.*, 2011; Zhao *et al.*, 2011; Nodine and Bartel, 2012). In addition, early reports suggest that dynamic changes in transcript proﬁles after fertilization are associated with *de novo* transcription and maternal elimination in the tobacco zygote (Zhao *et al.*, 2011), during which gamete functional specification, cytological elimination may also occur. However, whether cystatin family genes and the cysteine protease proteolytic pathway in which they participate are involved in these processes remains unknown. Thus, the expression level of cystatin family genes in sperm cells, egg cells, and zygotes of tobacco were quantified and compared in order to uncover the key genes of the cystatin family involved in sperm cell, egg cell, and zygote development.

**Fig. 6.**
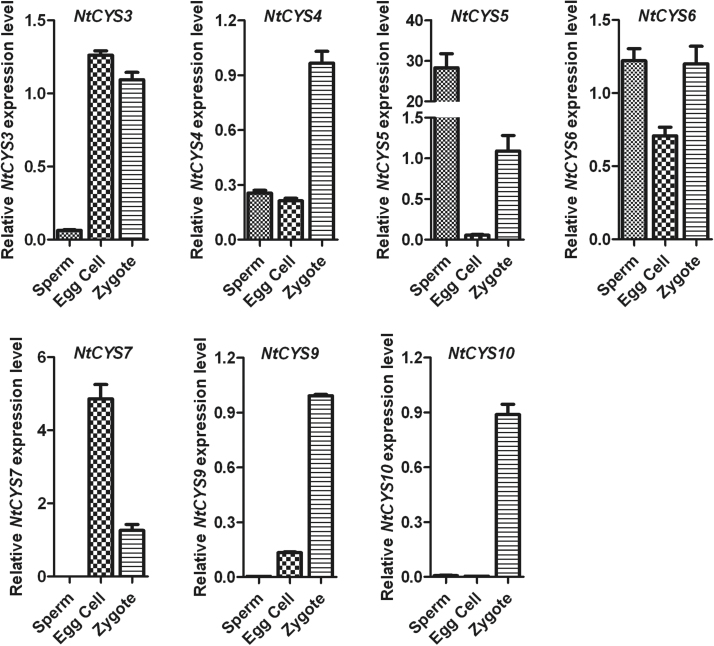
Relative expression levels of tobacco cystatin family genes in sperm cells, egg cells, and zygotes. The expression level of each cystatin in the zygote is set as 1. The expression level was normalized to the average expression level of GAPDH (AJ133422), polyubiquitin (GQ281244), and elongation factor 1α (AF120093). Error bars represent ±SE from three independent experiments.

The analysis showed that most of the tobacco cystatin genes can be detected in sperm, egg cells, and zygotes (Fig. 6). However, only one cystatin gene, *NtCYS5*, showed a significantly higher expression level in sperm cells (>20-fold more than the egg cell and zygote), suggesting that it may play important roles in sperm development. Cystatins in egg cells usually display a relatively lower level compared with those in sperm cells or zygotes, except for *NtCYS3* and *NtCYS7*. In contrast, most of the cystatins are abundant in zygotes compared with sperm cells and egg cells, with the exceptions of *NtCYS5* and *NtCYS7*. In addition, *NtCYS4*, *NtCYS9*, and *NtCYS10* show a significantly higher expression level in the zygote (>2-fold more than the sperm cell and egg cell). These results indicate that gamete-specific or preferential expression of cystatins exists in tobacco, and their differential expression probably plays distinct roles in gamete development and zygote formation.

### Zygotic asymmetric division results in uneven distribution of some cystatin transcripts in apical/basal cells

**Fig. 7.**
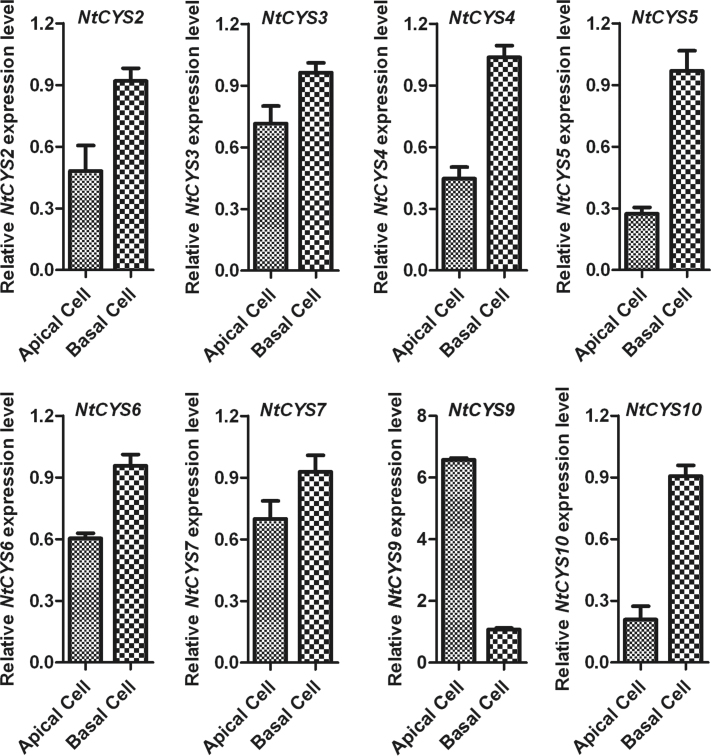
Relative expression levels of cystatin family genes in apical and basal cells. The expression level of each cystatin in the basal cell was set as 1. The expression level was normalized to the average expression level of GAPDH (AJ133422), polyubiquitin (GQ281244), and elongation factor 1α (AF120093). Error bars represent ±SE from three independent experiments.

The first asymmetric division of the one-celled proembryo (late zygote) is the beginning of sporophytic development in the plant, and usually gives rise to two daughter cells with distinct developmental fates. A small apical cell is the founder of a cell lineage generating the embryo proper, whereas a larger, basal cell establishes a cell lineage leading to the suspensor, which connects the embryo proper to maternal tissues (Goldberg *et al.*, 1994). Previous results of the analysis of the transcription profile showed that asymmetric zygotic division results in the uneven distribution of some specific embryogenesis-related transcripts in the two-celled proembryos (Ma *et al.*, 2011). From these findings, a basal suspensor cell-specific gene *NtCYS1*, which controls the onset of suspensor PCD by directly regulating the activity of the cathepsin H-like protease NtCP14, has been identified (Zhao *et al.*, 2013). To test whether the transcripts of other cystatin family genes also show similar uneven distribution patterns after asymmetric zygote division, the relative expression levels of the cystatin genes in apical and basal cells were quantified and compared with each other. The results indicate that only *NtCYS9* showed a significantly higher expression level in apical cells than in basal cells, suggesting that it may play specific roles in apical cell development. In contrast, the transcripts of most cystatin family genes are higher in basal cells than in apical cells (Fig. 7). Among them, two cystatin genes (*NtCYS5* and *NtCYS10*) showed significantly higher expression in basal cells (>2-fold), indicating that other cystatin family genes excluding *NtCYS1* may also function in basal cell differentiation and development.

### Most cystatins are present throughout the process of embryogenesis and seed formation

**Fig. 8.**
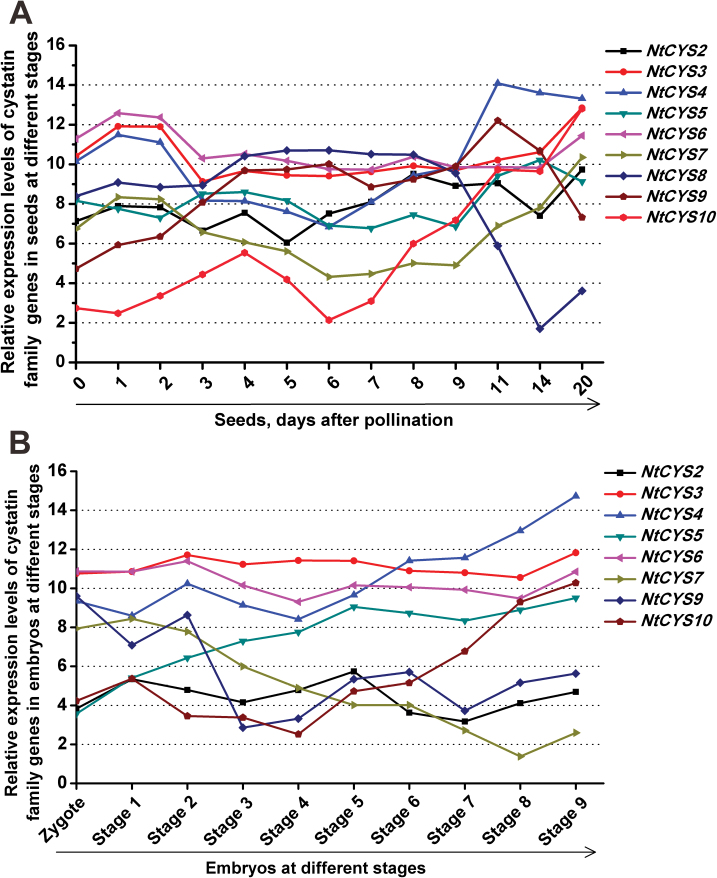
Transcript levels of cystatin family genes in seeds at different developmental stages. The expression level was normalized to the average expression level of GAPDH (AJ133422), polyubiquitin (GQ281244), and elongation factor 1α (AF120093). The data represent fold change (log2 value). (This figure is available in colour at *JXB* online.)

Flowering plant seeds build a highly elaborate functional unit with the aim of propagating offspring, which usually consists of integuments, embryo, and endosperm. From a descriptive point of view, plant seed formation can be divided into three major stages in which three distinct developmental and physiological events occur. The first stage is from immediately after fertilization to proembryo formation, the second is embryo transition, and the last is organ expansion and embryo maturation (Goldberg *et al.*, 1994). A previous expression pattern analysis of cystatin family genes in *H. vulgare* demonstrated that cystatin family genes participate in the regulation of seed germination (Martinez *et al.*, 2009). However, whether cystatin family genes are involved in embryogenesis and other stages of seed development still remains unknown. To identify important cystatins exclusively expressed in seed development, the transcript levels of each cystatin gene in seeds at successive developmental stages were detected and compared. The overview of cystatin family genes in the process of seed formation indicates that the majority of genes exhibit temporal and spatial variations in their expression pattern during the process of seed formation. The transcripts of all cystatin family genes in tobacco could be detected in seeds, but showed a different expression level at speciﬁc stages of seed development. Three expression peaks of cystatin family genes could be observed in the whole process of seed development, indicating that different cystatins are active at these stages (Fig. 8). The first expression peak was found in the process of fertilization, and the expression level of most cystains except *NtCYS5* in ovules was increased dramatically upon pollination (ovules at 1–2 d after pollination). The second expression peak was found after fertilization and during proembryo formation (seeds at 4–8 d after pollination). *NtCYS2*, *NtCYS4*, *NtCYS8*, *NtCYS9*, and *NtCYS10* showed peak expression during this process. The last expression peak was found at the stage of organ expansion and embryo maturation. The majority of cystatins except *NtCYS8* showed another expression peak at this stage.

The programme of embryogenesis plays a central role in deﬁning many of the key aspects of seed development. The process of embryo development in tobacco was classified into nine successive stages from the two-celled proembryo to the mature embryo, according to a previous report (Zhao *et al.*, 2013), and the expression profiles of cystatin family genes in embryos at stages 1–9 were built and compared with that of seeds at the corresponding stages. As shown in Fig. 8, most of the cystatin family genes can be detected in the embryos at different developmental stages. According to the characteristics of their expression pattern, they can be divided into three major groups. The first group comprise those whose transcription level is stable or shows no visible change during the whole process of embryogenesis. *NtCYS3* and *NtCYS6* belong to this group. The second group comprise those genes whose expression level increased gradually during the process of embryogenesis. *NtCYS4*, *NtCYS5*, and *NtCYS10* fall into this group. The third group consists of *NtCYS7* whose expression level decreased gradually during the process of embryogenesis. The transcription levels of other cystatin family genes show dynamic changes during the process of seed formation. All these data suggest that the different cystatin genes might collaborate with each other and play their different role in different stages as part of the network regulating embryogenesis and seed formation.

### Most cystatins are also expressed in the male reproductive organs

**Fig. 9.**
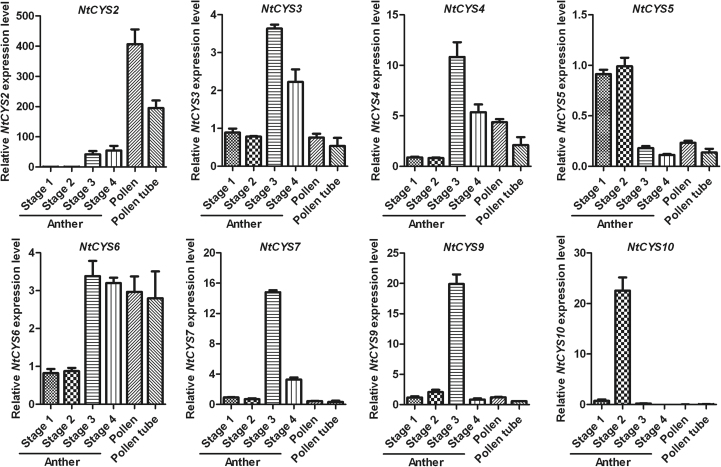
Transcript levels of cystatin family genes in male organs. The expression of each cystatin in anthers at stage 1 was set as 1. Anthers at stages 1–4 correspond to anthers containing microspore mother cells, tetrads, single-nucleated pollen, and bi-nucleated pollen, respectively. The expression level was normalized to the average expression level of GAPDH (AJ133422), polyubiquitin (GQ281244), and elongation factor 1α (AF120093). Error bars represent ±SE from three independent experiments.

Another striking feature of the expression profile of cystatin family genes is that several of them are abundant in male reproductive organs, especially in the anther at different stages (Figs 3, 9). They can be divided into two major groups according to the differences in the transcription level between the pollen and anther. Most of them show a higher expression level in the anther, but show a relatively lower expression level in pollen at the corresponding stage, indicating that cystatins in this group may be abundantly expressed in sporophytic tissue such as the tapetum. In addition, the transcription level of these genes shows dynamic changes during the process of anther development. Five of them (*NtCYS3*, *NtCYS4*, *NtCYS6*, *NtCYS7*, and *NtCYS9*) reach a peak when anthers develop into stage 3, and decrease gradually at later stages. Cystatin and their targets are known to be associated with various types of PCD (Solomon *et al.*, 1999; Belenghi *et al.*, 2003; van der Linde *et al.*, 2012), and their potential target, *OsCP1*, has been shown to be associated with tapetum PCD (Lee *et al.*, 2004; Li *et al.*, 2006). Therefore, these members may contribute to the regulation of tapetum PCD in tobacco. Conversely, another cystatin *NtCYS2* is abundant in pollen but lower in anther (>2-fold). The exact roles of *NtCYS2* in pollen development remain to be elucidated in a further study.

### Cystatins primarily inhibit the activities of cathepsin L-like proteases in early seeds

The expression profile analysis shows that the transcripts of most cystatin genes in tobacco can be detected in seeds at different stages and their transcription levels show dynamic changes during the whole process of seed development, suggesting that these members may have potential roles in seed development. To confirm the potential function of the proteolytic pathway of cystatin-dependent cysteine proteases in seed development, the inhibitory capabilities of each recombinant cystatin against different types of cysteine proteases in tobacco seeds were tested. Total proteins were extracted from seeds at stage 1, 3, 6, 7, and 9, respectively. Each cystatin was then used to inhibit proteolytic activities in extracts from tobacco seeds at different stages, using substrates Z-FR-AMC (for cathepsin L-like proteases), Z-RR-AMC (for cathepsin B-like proteases), and Bz-FVR-AMC (for cathepsin H-like proteases), respectively. The assays were carried out by adding 1 μM of each cystatin to 5 μg of soluble protein extracts from seeds. The results indicated that each cystatin inhibited primarily the activities of cathepsin L-like proteases from early seeds at stages 1–6, and then the activities of cathepsin H-like proteases (Table 3; Supplementary Table S3 at *JXB* online). Each recombinant cystatin showed a signiﬁcantly reduction (by ~90.8–98%) in the cathepsin L-like activities present in tobacco seeds at stages 1–6, and cathepsin H-like activities were also inhibited by some cystatins (by ~76.7–41.5%), whereas only a few cystatins can inhibit cathepsin B-like activities (by ~13.3–42.4%). In contrast, when embryos develop into stage 7, the inhibitory potency of each cystatin against cathepsin L-like activities decreased significantly (by ~14.3–29.7%). Similar to the inhibitory potency against cathepsin L-like activities, the inhibitory potency of each cystatin against cathepsin B- and H-like activity also decreased significantly. All these data suggest that cystatin-dependent cathepsin L-like proteolytic pathways are important for early seed development.

**Table 3.** Inhibitory activities of the recombinant cystatins against total proteins from seeds at different stagesData are expressed as percentages compared with their corresponding control without recombinant cystatin. No inhibitory effect (Ni) was considered for inhibition <10% at 1 μM of each recombinant cystatin (*n*=3).

Cystatin	Stage 1			Stage 3			Stage 6			Stage 7			Stage 9		
	FR-AMC	RR-AMC	FVR-AMC	FR-AMC	RR-AMC	FVR-AMC	FR-AMC	RR-AMC	FVR-AMC	FR-AMC	RR-AMC	FVR-AMC	FR-AMC	RR-AMC	FVR-AMC
NtCYS2	4.5±0.2	73.0±3.3	37.3±2.7	2.2±0.5	62.5±5.4	34.1±4.6	7.8±4.6	73.7±10.1	46.7±4.9	81.6±1.5	86.0±2.2	75.7±4.9	78.2±4.3	Ni	79.1±2.6
NtCYS3	4.1±0.2	63.0±2.3	24.8±1.8	2.7±0.5	58.6±3.6	36.3±5.1	7.6±4.8	71.1±8.1	42.1±3.7	73.9±3.4	84.4±2.0	64.9±3.5	70.3±2.5	88.5±1.4	70.9±1.7
NtCYS4	4.2±0.2	61.5±2.1	25.1±2.3	2.1±0.4	57.6±5.2	35.0±3.9	7.2±3.8	69.5±10.0	42.9±4.0	76.6±1.6	82.2±1.5	67.4±3.7	74.9±2.2	86.3±1.8	74.1±3.1
NtCYS5	4.4±0.2	65.3±1.5	24.9±3.2	2.1±0.5	58.6±5.1	26.4±4.7	7.5±4.0	70.4±10.0	42.8±3.2	77.2±2.1	83.3±2.7	70.0±4.6	75.0±2.9	86.7±1.5	76.4±2.3
NtCYS6	4.7±0.3	86.7±1.7	44.2±3.0	2.3±0.4	79.0±8.0	37.6±5.2	7.3±4.2	76.9±7.9	52.4±6.7	82.7±3.1	Ni	85.7±3.6	78.4±3.1	Ni	85.0±2.1
NtCYS7	5.9±0.7	Ni	51.6±3.4	4.0±1.2	Ni	51.8±6.5	9.2±5.1	86.2±5.5	59.5±7.5	85.7±2.0	Ni	Ni	79.6±3.7	Ni	Ni
NtCYS8	Ni	Ni	Ni	Ni	Ni	Ni	Ni	Ni	Ni	Ni	Ni	Ni	Ni	Ni	Ni
NtCYS9	4.0±0.3	62.5±2.3	30.6±1.5	2.0±0.4	55.1±4.1	23.3±4.2	6.7±3.7	67.8±9.4	39.8±5.8	74.8±1.5	81.6±1.4	69.3±3.5	72.5±2.3	84.6±1.4	73.4±2.0
NtCYS10	4.2±0.2	65.0±1.9	27.2±2.1	2.2±0.5	59.5±5.6	32.1±3.1	7.1±3.8	71.6±8.1	44.8±4.0	75.1±2.6	80.2±1.5	66.1±4.1	71.7±3.4	84.6±2.2	72.1±2.0

### Intracellular localization of cystatins

**Fig. 10.**
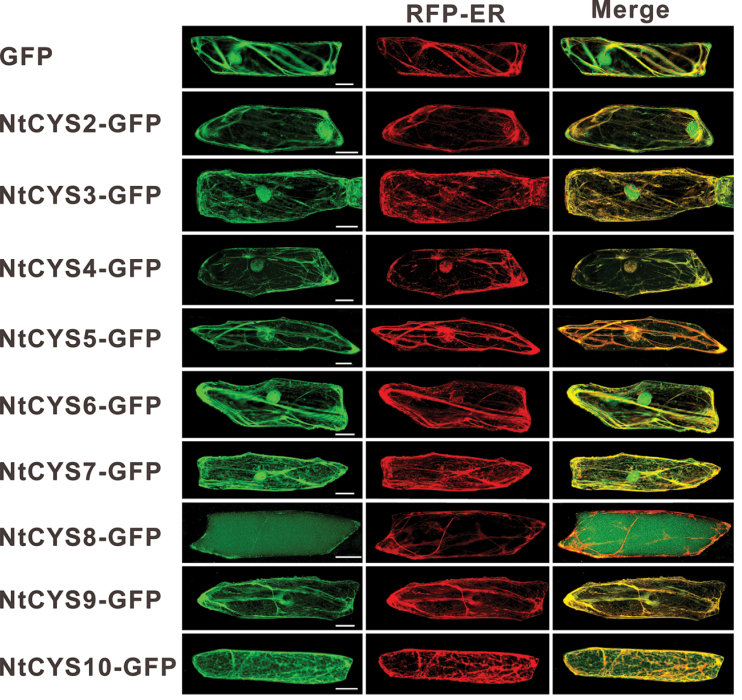
Intracellular localization of tobacco cystatins in *A. cepa* epidermal cells. GFP alone was used as a control. Scale bars = 50 μm. (This figure is available in colour at *JXB* online.)

To gain insight into the intracellular localization of the cystatins in tobacco, the ORFs of the cystatin genes were cloned into a pRS300 fused to eGFP and driven by the 35S promoter. Fluorescent proteins were transiently expressed in the epidermal cells of *A. cepa*, and it was found that most of the cystatins are recognized as secretory proteins as they contain signal peptides (Table 1). The signal peptide is known to direct them into the ER finally to be targeted to different destinations from the ER. An earlier report proved that NtCYS1 can co-localize with an ER marker, implying that signal peptides of NtCYS1 can direct it to the ER. In the present work, it was found that other cystatins with signal peptides could also enter the ER, and were finally targeted to different compartments. Among them, NtCYS8 was targeted to the vacuole from the ER, and some other cystatins always have strong co-localization with an ER marker (Fig. 10) (Haseloff *et al.*, 1997), but not with a Golgi marker (Saint-Jore *et al.*, 2002) in *A. cepa* epidermal cells (Supplementary Fig. S3 at *JXB* online); whereas two cystatins (NtCYS6 and NtCYS7) without signal peptides showed similar distributions to GFP alone. They could be detected in both the cytoplasm and nucleus. Surprisingly, two other cystatins (NtCYS2 and NtCYS3) with signal peptides were also targeted to the nucleus like HvCPI-1 and HvCPI-4 reported in *H. vulgare* (Martinez *et al.*, 2009).

## Discussion

### Main characteristics of cystatin family genes in tobacco

A considerably large amount of sequence data of cystatin family genes from different species is currently available in GenBank, including some fully sequenced species. Seven cystatin genes in *A. thaliana* and 12 in *O. sativa* have been predicted. However, only one cystatin gene, *NtCYS1*, was cloned from *N. tabacum* (Zhao *et al.*, 2013). *Nicotiana tabacum*, a traditional model plant, is assumed to originate from a hybridization event between ancestors of *N. sylvestris* and *N. tomentosiformis* ~200 000 years ago (Sierro *et al.*, 2013b). The draft genomes of *N. sylvestris* and *N. tomentosiformis* have been sequenced and assembled (Sierro *et al.*, 2013a). The available draft genomes of *N. sylvestris* and *N. tomentosiformis*, as well as ESTs covering different cDNA libraries, especially cell-type specific cDNA libraries of sperm, egg cells, zygotes, and early embryos, have been constructed, which facilitates the identification of cystatin family genes in tobacco. In the present study, 10 cystatin genes were identified and divided into three groups based on phylogenetic analysis. Some conserved motifs for the cystatin family have been identified through the alignment of the amino acid sequences. Apart from the ‘LARFAV’ motif with some substitutions, most of the cystatins, with the exception of NtCYS1 and NtCYS8, contain three core motifs forming the tripartite wedge that enters the active site responsible for inhibiting their targets (Bode *et al.*, 1988; Arai *et al.*, 1991). The central ‘QxVxG’ motif is important for the inhibition process since it can directly enter and interact with the active site of targeted enzymes, which was proved by the comparison of the inhibitory capacity of a direct mutation of the ‘QxVxG’ region of rice oryzacystatin OC-I toward papain (Arai *et al.*, 1991). In the present study, the central ‘QVVATG’ instead of ‘QxVxG’ was found in NtCYS8, which showed no visible inhibitory potency for the four cysteine proteases tested. Moreover, recombinant NtCYS8 also lacked the inhibitory effect against protein extracts from tobacco seeds, confirming that the typical ‘QxVxG’ motif is essential for the process of inhibition. Similarly, two other elements, G in the N-terminus and W in the C-terminus also seem to be important for the inhibitory capability as shown by the *K*_i_ values for different cysteine proteases. In addition, another two conserved motifs, ‘VWxKPW’ and ‘KxLxxF’, were detected in the C-terminus of all cystatins. However, the precise connection between these motifs and the inhibitory capability for cathepsin-like proteases remains to be elucidated in a further study.

### Tobacco cystatins probably play significant roles in gamete development, embryogenesis, and seed development

Several physiological functions of cystatin genes in plants have been demonstrated, such as PCD (Solomon *et al.*, 1999; Zhao *et al.*, 2013), seed germination (Hwang *et al.*, 2009), and defence against biotic and abiotic environmental stresses (Hwang *et al.*, 2010). However, whether cystatin family genes are involved in other processes of plant development, especially in sexual plant reproduction, has attracted a great deal of attention but still remains to be explored. The characterization of cystatin genes in *H. vulgare* suggested their potential roles in hordein mobilization during seed germination (Martinez *et al.*, 2009). In order to survey the putative functions of cystatin family genes in plants, it is essential to analyse extensively their expression pattern, subcellular location, and inhibitory potency against different types of cysteine proteases. The present data reveal temporal and spatial characters of the expression of these cystatin genes in tobacco. Interestingly, it was found that the transcripts of most cystatins can be detected in male and female gametes, which suggests that cystatin family genes may have other specific uncharacterized roles in gamete development. It is well known that gamete structure and functional specification is critical for fertilization in animals. Although whether plant gametes undergo a similar process during their development remains unclear, cytoplasm reorganization or organelle deletion in this process has at least been reported in male gametes (Dickinson and Grant-Downton, 2009; Berger and Twell, 2011). The role of cystatins in this critical process will be a novel field of study for researchers.

Another interesting finding is that the transcription of the majority of cystatin genes is spatially regulated in the processes of embryo development and seed formation. Three of the cystatins were preferentially expressed in seeds (Fig. 5). In addition, most recombinant cystatins (except NtCYS8) have the ability to inhibit the activities of model cysteine proteases in the peptidase C1A family in extracts from tobacco seeds *in vitro*, especially the cathepsin L-like proteases in early seeds. It is thus proposed that cystatins in tobacco may have potential roles in seed development, especially in early seed development. The spatial regulation of the expression of these cystatins may be coupled with specific developmental events during early embryogenesis and seed formation, and this is worthy of further study.

### Mechanism of cystatin regulating the activities of cysteine proteases

The activities of papain-like proteases may be controlled by several different mechanisms including local zymogen concentration and the presence of a specific repertoire of inhibitors, as suggested in a recent study (Cambra *et al.*, 2012). Cystatins are tightly bound and reversible inhibitors of cysteine proteases; some of them have been shown to have the capacity to inhibit the activities of papain-like proteases (Arai *et al.*, 2002), and a few of them can also inhibit the activities of legumain-like protease (Martinez *et al.*, 2007). In the present study, 10 cystatin family genes in tobacco have been identified and characterized intensively, and nine of them can primarily inhibit the activities of both cathepsin L and cathepsin L-like proteases in seeds *in vitro*. However, how they regulate the activities of cysteine proteases *in vivo* still needs to be explored. Here, it is proposed that cystatin may regulate the activities of their targeted proteases in three ways, namely the transcriptional regulation of cystatin family genes in different tissues, the intracellular compartmentalization of cystatins, and via the specific-motifs in cystatins responsible for their inhibitory potency.

Transcriptional regulation of gene expression is thought to be a primary mechanism responsible for their expression pattern in different tissues, and this been shown to be controlled by a set of transcriptional factors. Here, expression profile analysis of the cystatin family genes in tobacco revealed that cystatins showed a wide diversity of expression patterns, implying a functional diversity of all members of the cystatin family genes in regulation of the activities of cysteine proteases in a tissue- or stage-specific manner during seed development. To date, the transcription factor that regulates the expression of cystatin family genes has not yet been identified. It was reported that a putative basic helix–loop–helix transcriptional factor TDR could regulate the expression of *OsCP1*, a potential target of rice cystatin, in tapetum (Li *et al.*, 2006). Generally, most of the cystatins are recognized as secretory proteins, as they contain signal peptides that direct them into the ER and finally to different destinations for binding to target proteases and inhibiting their activities in a specific intracellular site. However, the exact location of the cystatin family proteins in a cell and their compartment-specific targets have not been characterized in plants. In addition, the mechanism for cystatin regulation of the activities of their target proteases might be based on the specific motif in cystatin. A single amino acid substitution in the eighth domain of tomato cystatin SlCYS8 exhibited either improved or lowered potency against different model cysteine proteases, suggesting the specific amino acids in cystatin as target sites to regulate the inhibitory potency of the cystatin (Goulet *et al.*, 2008). Improved binding properties of cystatins with selected site mutations were demonstrated in site-directed mutagenesis of LeCYS8 (Kiggundu *et al.*, 2006). In addition, the ‘SNS’ motif in cystatin was shown to be essential for inhibiting the activities of legumain-like proteases in both animals and plants (Alvarez-Fernandez *et al.*, 1999; Martinez *et al.*, 2007). In the present study, comprehensive inhibitory potency analysis of all cystatins in tobacco against different types of cysteine proteases *in vitro* showed different *K*_i_ values of cystatins for their targeted cysteine proteases, indicating their preferential inhibitory capacity for their specific targets. A search for positively selected residues of cystatins will surely facilitate understanding of the regulatory mechanism between plant cystatins and their targeted enzymes *in vivo*.

### Accession numbers

Sequence data for the cystatin family genes in tobacco can be found in GenBank (http://www.ncbi.nlm.nih.gov/Genbank) under the following accession numbers: NtCYS1 (KF113570), NtCYS2 (KJ725113), NtCYS3 (KJ725114), NtCYS4 (KJ725115), NtCYS5 (KJ725116), NtCYS6 (KJ725117), NtCYS7 (KJ725118), NtCYS8 (KJ725119), NtCYS9 (KJ725120), and NtCYS10 (KJ725121).

## Supplementary data

Supplementary data are available at *JXB* online.

Figure S1. Sequence alignment of cystatin protein sequences in tobacco.

Figure S2. Predicated three-dimensional structures of cystatins in tobacco.

Figure S3. Intracellular localization of tobacco cystatins in *A. cepa* epidermal cells.

Table S1. Primers used in RT–PCR and RT–qPCR.

Table S2. Primers used in vector construction.

Table S3. Protease activities in extracts from developing tobacco seeds with or without recombinant cystatins.

Supplementary Data

## Abbreviations:

ER: endoplasmic reticulum
ORF: open reading frame
PCD: programmed cell death
RACE: rapid amplification of cDNA ends
RT–PCR: reverse transcription–PCR
RT–qPCR: quantitative real-time reverse transcription–PCR.
