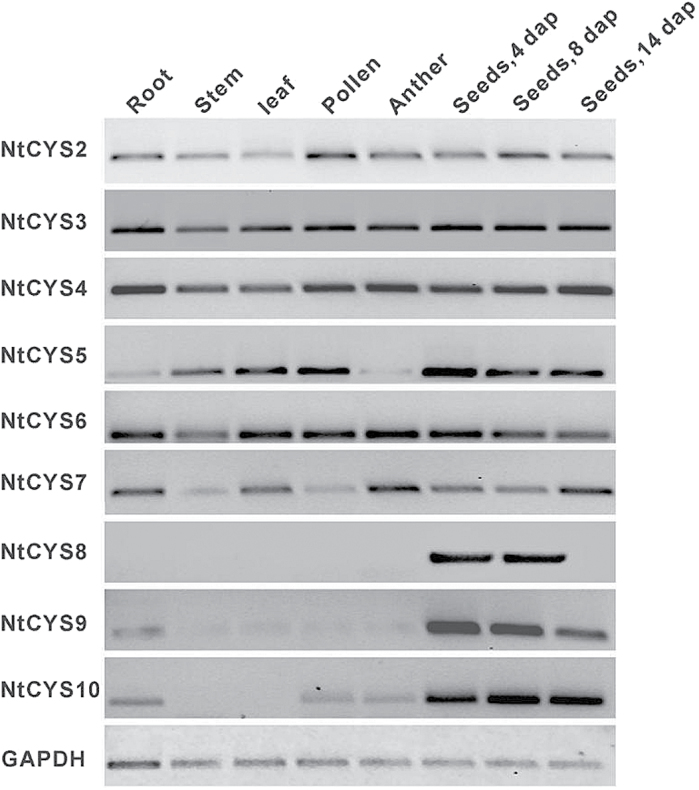# Corrigendum

**DOI:** 10.1093/jxb/erv411

**Published:** 2015-08-27

**Comprehensive analysis of cystatin family genes suggests their putative functions in sexual reproduction, embryogenesis, and seed formation**

Peng Zhao^1^,*, Xue-mei Zhou^1^,*, Jie Zou^2^, Wei Wang^1^, Lu Wang^3^, Xiong-bo Peng^1^ and Meng-xiang Sun^1^,†

^1^ Department of Cell and Developmental Biology, College of Life Sciences, State Key Laboratory of Plant Hybrid rice, Wuhan University, Wuhan 430072, China

^2^ Molecular Genetics Key Laboratory of China Tobacco, Guizhou Academy of Tobacco Science, Guiyang 550081, China

^3^ Key Laboratory of Plant Germplasm Enhancement and Specialty Agriculture, Wuhan Botanical Garden of the Chinese Academy of Sciences, Wuhan 430074, China

* These authors contributed equally to this work.

† To whom correspondence should be addressed. E-mail: mxsun@whu.edu.cn

*Journal of Experimental Botany*, Vol. 65, No. 17, pp. 5093–5107, 2014, doi:10.1093/jxb/eru274

In Figure 1 of the above paper, the image for CYS2 was inadvertently used to represent the expression pattern of both CYS2 and CYS3. The correct image for CYS3 is provided in the below revised version of this figure:

**Figure:**