# Supplementary material for: Comprehensive analysis of cystatin family genes suggests their putative functions in sexual reproduction, embryogenesis, and seed formation
Source: J Exp Bot. 2014 Jul 4;65(17):5093–107. doi: 10.1093/jxb/eru274 (PMC4144781; doi:10.1093/jxb/eru274)
Supplement: Supplementary Data [file supp_65_17_5093__index.html]

Comprehensive analysis of cystatin family genes suggests their putative functions in sexual reproduction, embryogenesis, and seed formation — Comprehensive analysis of cystatin family genes suggests their putative functions in sexual reproduction, embryogenesis, and seed formation — Supplementary Data 

# Comprehensive analysis of cystatin family genes suggests their putative functions in sexual reproduction, embryogenesis, and seed formation

## Supplementary Data

Data files

**Files in this Data Supplement:**

- Supplementary Data - Supplementary Data
