# Supplementary material for: Comprehensive analysis of cystatin family genes suggests their putative functions in sexual reproduction, embryogenesis, and seed formation
Source: J Exp Bot. 2014 Jul 4;65(17):5093–107. doi: 10.1093/jxb/eru274 (PMC4144781; doi:10.1093/jxb/eru274)
Supplement: Supplementary Data [file supp_eru274_jexbot123224_file001.pdf]

Comprehensive analysis of cystatin family genes suggests their putative functions in sexual reproduction, embryogenesis and seed formation

Peng Zhao, Xue-mei Zhou, Jie Zou, Wei Wang, Lu Wang, Xiong-bo Peng, Meng-xiang Sun

Supplemental material

The supplemental material includes three figures and three tables.

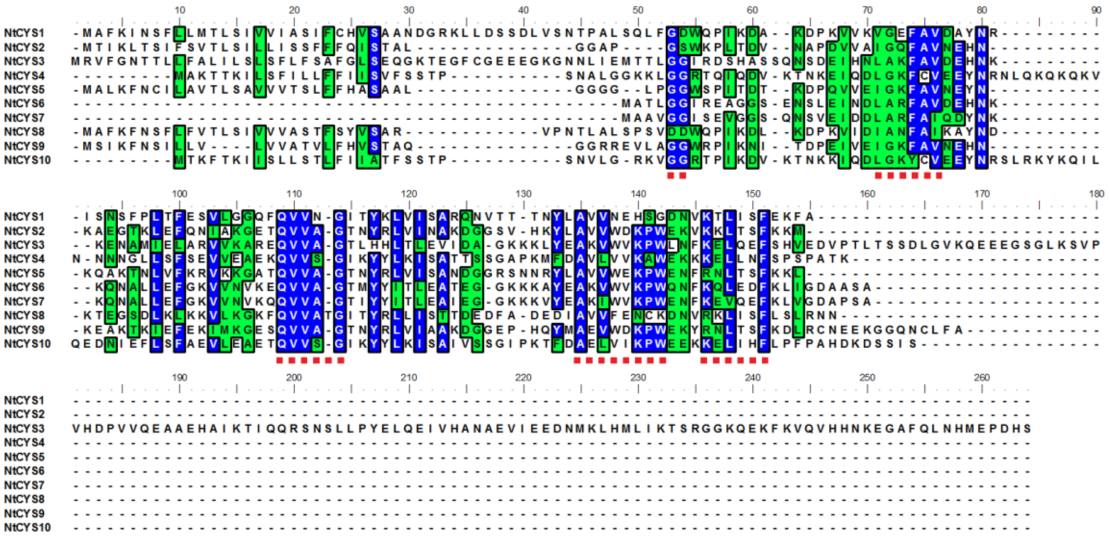

Fig. S1. Sequence alignment of cystatin protein sequences in tobacco

Identical residues are outlined and shaded blue. Similar residues are outlined and shaded green.

Conserved motifs in cystatins are labeled with red-dot lines.

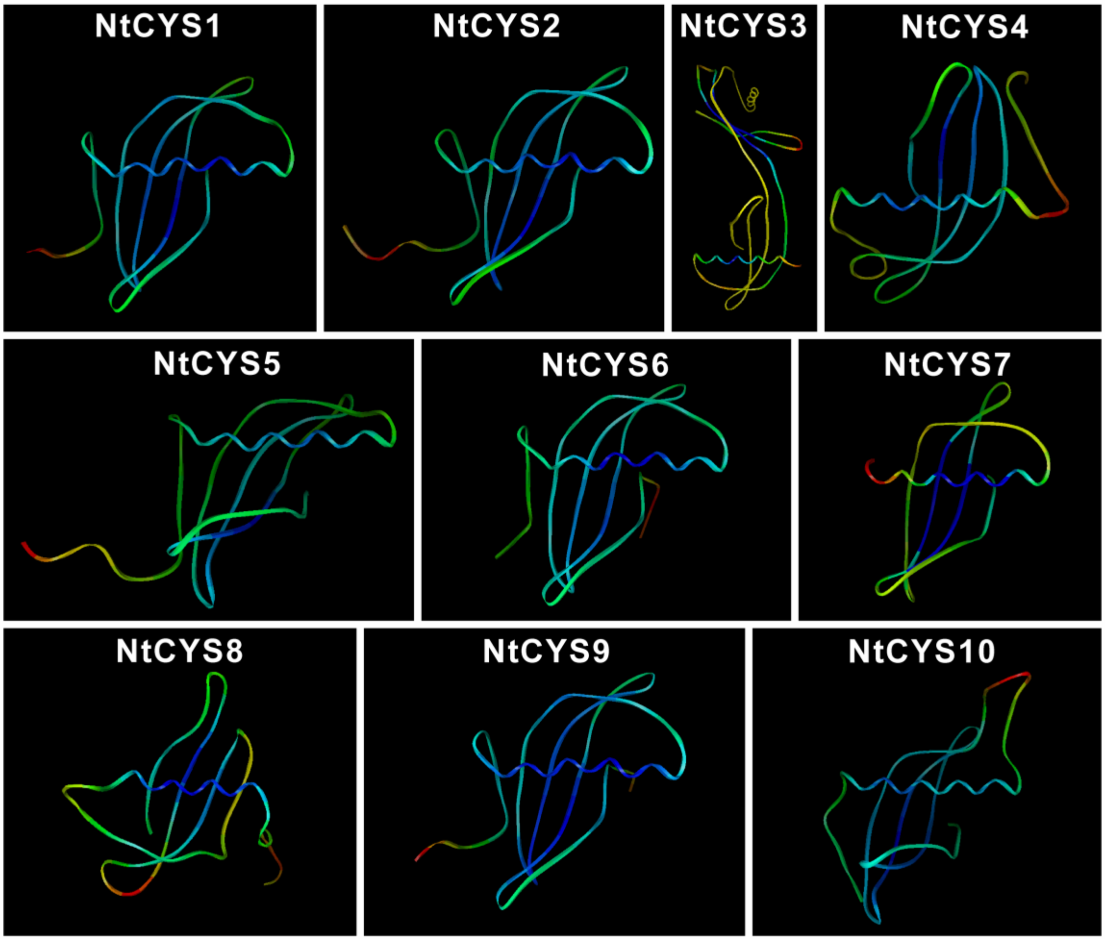

Fig. S2. Predicated three-dimensional structures of cystatins in tobacco

Three-dimensional structures of cystatin proteins in tobacco are colored according to 3D Molecular Viewer temperature color scheme. The color codes each atom according to the anisotropic temperature value stored in the PDB file. High values are colored in warmer colors (red) and lower values in colder colors (blue).

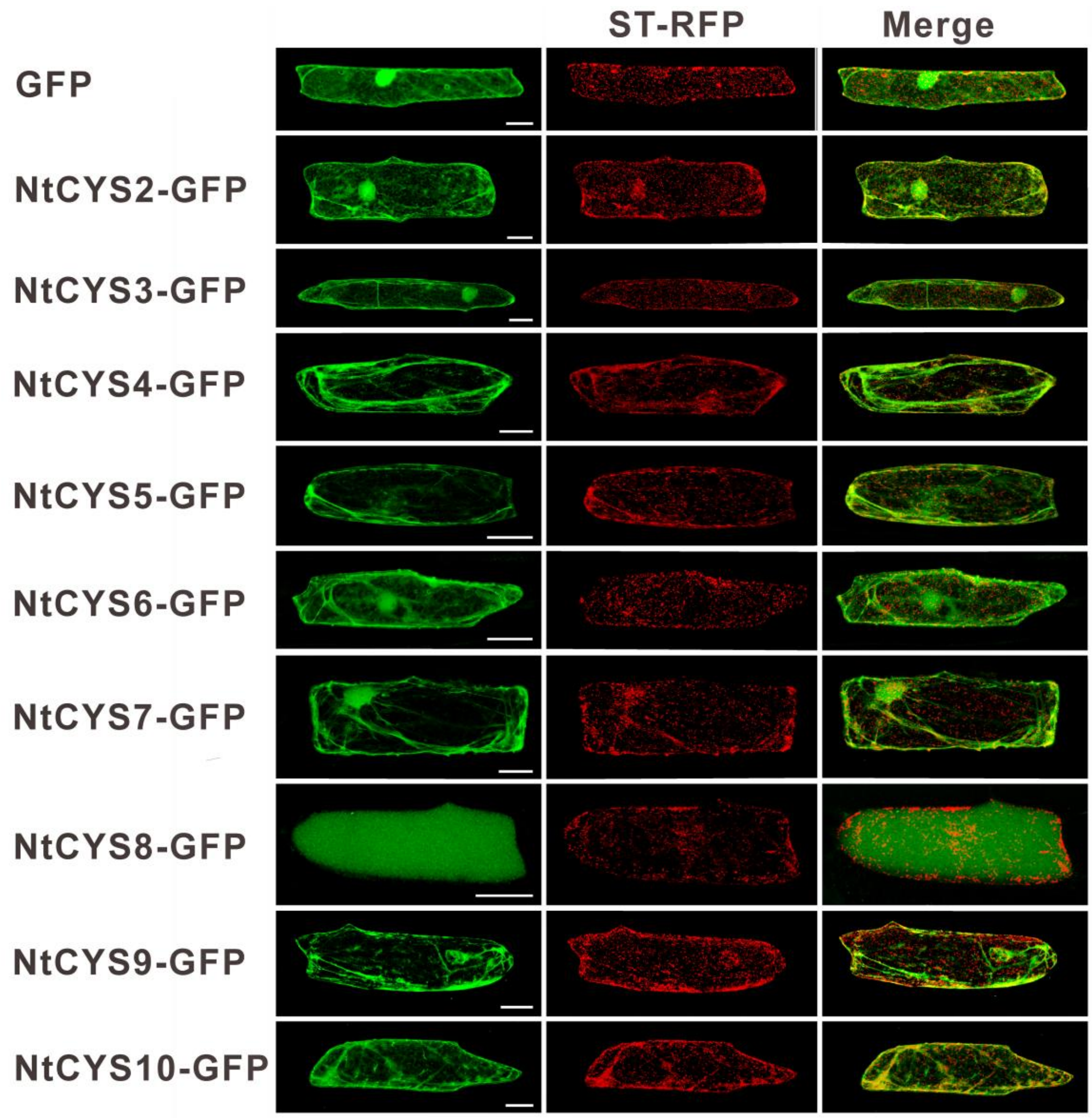

Fig. S3. Intracellular localization of tobacco cystatins in *A. cepa* epidermal cells

GFP alone was used as the control. Scale bars, 50 μm.

Table S1. Primers used in RT-PCR and RT-qPCR

Cystatins	For RT-PCR		For RT-qPCR	
	Forward	Reverse	**Forward**	**Reverse**
NtCYS2	CACAATAAAGCGGAAGGGACAA	ATCAACGAGGCGGATCTAGGAC	TCGTCCTAGATCCGCCTCGT	CCAACATCACATTTTCTCATTTTTTC
NtCYS3	GATTCGTGATTCTCATGCTTCGTCC	CCCAAACCTTAGCCTCATAGAGTTTCTT	ACTACTCTTGGTGGGATTCGTG	TTCTCCTTCTTGTTGTGCTCG
NtCYS4	TAAAATCTCCGCAACTACTTCCTC	GTCACCCAAAATACGTTACAAATCA	TGGGAAAGTTTTGTGTTGAAGAG	CCTGAGGAAGTAGTTGCGGAG
NtCYS5	GTCACCTCCCTCTTCTTCCACGCCT	CCAAGTACCTATTGTTGCTACGACCACC	GGTCACCTCCCTCTTCTTCCA	GCTCCCTTCTTCACTCGTTTG
NtCYS6	TCTTGCTCGCTTTGCTGTTG	ACCTTGGCTTCGTATGCTTTCT	TCTTGCTCGCTTTGCTGTTG	CCTTGGCTTCGTATGCTTTCT
NtCYS7	TGAGGGCGGAAAGAAGAAAG	CAAATACACTAGGAGCAGAAAACCA	ATGATCTTGCACGTTTTGCTATC	TACTTTCTTCTTTCCGCCCTC
NtCYS8	TTGCTTCCACCTTCTCCTATG	CAAAAACAACCGCTATATCCTC	CCGTCAGTTGATGATTGGCA	ATCTTCGTCCGTGGTGGAAA
NtCYS9	CCGTTCTCTTCCATGTCTCCACC	GGCTCTCTCCCTTCATTATTTTCTCAA	GGTTGCCACCGTTCTCTTCC	CTCAGGGTCCGTTATGTTTTTTATG
NtCYS10	ACTCTCTTCATAATAGCAACATTTTCTTCAACCC	GCGGAGATTTTAAGGTAATATTTGATCCCA	TCATTTCCCTTCTCTCCACTCTC	CTTCCCCCAACTTTTCGTCC

Table S2. Primers used in vector construction

Cystatin	For pMXB10	
	Forward	Reverse
NtCYS2	NNNN CATATG TTGGGCGGCGCACCAGGTAGTTGGAAG	NNNN CTCGAG CATTTTTTTGAAAGAAGTAAGCTTC
NtCYS3	NNNN CCATGG AT TTAAGCGAACAAGGGAAAACCG	NNNN CTCGAG GGAGTGGTCAGGCTCCATATGATTC
NtCYS4	NNNN CATATG TCAACCCCATCAAATGCTTTAGG	NNNN CTCGAG CTTAGTAGCAGGAGAAGGAGAAAAG
NtCYS5	NNNN CATATG CTCGGCGGTGGTGGTTTACCAG	NNNN CTCGAG TAATTTTTTGAAGGAAGTGAGGTTCC
NtCYS6	NNNN CATATG ATGGCAACTCTAGGAGGAATTC	NNNN CTCGAG AGCACTAGCGGCATCCCCAATAAGC
NtCYS7	NNNN CATATG ATGGCAGCTGTAGGAGGCATTAG	NNNN GCGGCCGC C AGCACTTGGGGCATCGCCAAC
NtCYS8	NNNN CATATG CGCGTACCGAATACTCTAGCTCTG	NNNN CTCGAG ATTGTTACGCAAACTTAAAAAAG
NtCYS9	NNNN CATATG CAGGGCGGTCGGAGAGAGGTTTTG	NNNN CTCGAG AGCAAAAAGACAATTTTGTCCTCC
NtCYS10	NNNN CATATG CGAAAAGTTGGGGGAAGAAC	NNNN CTCGAG ACTAATACTACTGTCTTTATCATG
Cystatin	For pRS300-35S-eGFP-NOS	
	Forward	Reverse
NtCYS2	NNNN TCTAGA ATGACCATCAAATTGACCTCTATTTTC	NNNN CTGCAG CATTTTTTTGAAAGAAGTAAGC
NtCYS3	NNNN GAATTC ATGAGAGTATTTGGTAACACCACAC	NNNN CTCGAG GGAGTGGTCAGGCTCCATATGATTC
NtCYS4	NNNN TCTAGA ATGGCAAAAACAACAAAAATCCTC	NNNN CTGCAG CTTAGTAGCAGGAGAAGGAGAAAAG
NtCYS5	NNNN TCTAGA ATGGCCCTCAAATTCAACTGTATCC	NNNN CTGCAG TAATTTTTTGAAGGAAGTGAGGTTCC
NtCYS6	NNNN TCTAGA ATGGCAACTCTAGGAGGAATTCGTG	NNNN CTGCAG AGCACTAGCGGCATCCCCAATAAGCTTG
NtCYS7	NNNN TCTAGA ATGGCAGCTGTAGGAGGCATTAGC	NNNN CTGCAG AGCACTTGGGGCATCGCCAACAAG
NtCYS8	NNNN TCTAGA ATGGCTTTCAAATTCAATTCTTTTCTTTTTG	NNNN CTGCAG ATTGTTACGCAAACTTAAAAAAG
NtCYS9	NNNN TCTAGA ATGTCTATCAAATTCAATTCCATCCTC	NNNN CTGCAG AGCAAAAAGACAATTTTGTCCTCC
NtCYS10	NNNN TCTAGA ATGACGAAATTCACAAAAATCATTTC	NNNN CTGCAG ACTAATACTACTGTCTTTATCATG

Table S3. Protease activities in extracts from developing tobacco seeds with or without recombinant cystatins

Cystatin	Stage 1			Stage 3			Stage 6			Stage 7			Stage 9		
	FR-AMC	RR-AMC	FVR-AMC	FR-AMC	RR-AMC	FVR-AMC	FR-AMC	RR-AMC	FVR-AMC	FR-AMC	RR-AMC	FVR-AMC	FR-AMC	RR-AMC	FVR-AMC
	V0 (μM per hour per μg protein)			V0 (μM per hour per μg protein)			V0 (μM per hour per μg protein)			V0 (μM per hour per μg protein)			V0 (μM per hour per μg protein)		
CK	32.02±2.86	2.55±0.08	3.61±0.13	38.83±4.39	1.47±0.05	2.86±0.37	5.95±1.58	0.60±0.15	0.75±0.28	0.99±0.06	1.13±0.13	0.84±0.07	1.08±0.05	1.03±0.11	0.77±0.07
NtCYS2	1.44±0.06	1.86±0.08	1.35±0.10	0.85±0.19	0.92±0.08	0.98±0.13	0.46±0.27	0.44±0.06	0.35±0.04	0.81±0.01	0.97±0.02	0.64±0.04	0.84±0.05	0.94±0.01	0.61±0.02
NtCYS3	1.31±0.06	1.61±0.06	0.90±0.06	1.05±0.19	0.86±0.05	1.04±0.15	0.45±0.29	0.43±0.05	0.32±0.03	0.73±0.03	0.95±0.02	0.55±0.03	0.76±0.03	0.91±0.01	0.55±0.01
NtCYS4	1.34±0.06	1.57±0.05	0.91±0.08	0.82±0.16	0.85±0.08	1.00±0.11	0.43±0.23	0.42±0.06	0.32±0.03	0.76±0.02	0.93±0.02	0.57±0.03	0.81±0.02	0.89±0.02	0.57±0.02
NtCYS5	1.41±0.06	1.67±0.04	0.90±0.12	0.82±0.19	0.86±0.07	0.76±0.13	0.45±0.24	0.42±0.06	0.32±0.02	0.76±0.02	0.94±0.03	0.59 ±0.04	0.81±0.03	0.89±0.02	0.59±0.02
NtCYS6	1.50±0.10	2.21±0.04	1.60±0.11	0.89±0.16	1.16±0.12	1.08±0.15	0.43±0.25	0.46±0.05	0.39±0.05	0.82±0.03	1.03±0.02	0.72±0.03	0.85±0.03	0.99±0.01	0.65±0.02
NtCYS7	1.89±0.22	2.41±0.11	1.86±0.12	1.55±0.47	1.32±0.14	1.48±0.19	0.55±0.30	0.52±0.03	0.45±0.06	0.85±0.02	1.05±0.05	0.77±0.03	0.86±0.04	0.99±0.02	0.69±0.01
NtCYS8	30.06±1.95	2.53±0.11	3.36±0.16	36.85±3.61	1.40±0.09	3.00±0.23	5.81±0.79	0.55±0.02	0.70±0.04	0.98±0.03	1.06±0.03	0.79±0.02	1.00±0.04	0.97±0.01	0.70±0.02
NtCYS9	1.28±0.10	1.59±0.06	1.10±0.05	0.78±0.16	0.81±0.06	0.67±0.12	0.40±0.22	0.41±0.06	0.30±0.04	0.74±0.01	0.92±0.02	0.58±0.03	0.78±0.02	0.87±0.01	0.57±0.02
NtCYS10	1.34±0.06	1.66±0.05	0.98±0.08	0.85±0.19	0.87±0.08	0.92±0.09	0.42±0.23	0.43±0.05	0.34±0.03	0.74±0.03	0.91±0.02	0.56±0.03	0.77±0.04	0.87±0.02	0.56±0.02

CK means the hydrolytic activities of cysteine proteases in seeds at different stages without addition of any recombinant cystatins. (n=3)
